# Cognition and Frailty in Patients With Heart Failure: A Systematic Review of the Association Between Frailty and Cognitive Impairment

**DOI:** 10.3389/fpsyt.2021.713386

**Published:** 2021-07-02

**Authors:** Kenneth M. Faulkner, Izabella Uchmanowicz, Magdalena Lisiak, Ewelina Cichoń, Tomasz Cyrkot, Remigiusz Szczepanowski

**Affiliations:** ^1^Stony Brook University School of Nursing, Stony Brook, New York, NY, United States; ^2^Department of Clinical Nursing, Faculty of Health Sciences, Wroclaw Medical University, Wroclaw, Poland; ^3^Department of Psychology, WSB University in Torun, Torun, Poland; ^4^Department of Psychology, Faculty of Applied Studies, University of Lower Silesia, Wroclaw, Poland; ^5^Department of Public Health, Faculty of Health Sciences, Wroclaw Medical University, Wroclaw, Poland

**Keywords:** cognition, frailty, heart failure, review, cognitive impairment

## Abstract

**Background/Aim:** Pathological processes associated with aging increase the risk of cognitive deficits. Frailty syndrome may significantly accelerate these pathological processes in elderly patients with heart failure. The objective of this review was to better understand the association between frailty syndrome and co-occurring cognitive decline in patients with heart failure.

**Methods:** We conducted a systematic review based on PubMed/MEDLINE, Scopus, EMBASE, and CINAHL as databases. The search followed the method described by Webb and Roe. For inclusions, the studies were selected employing cross-sectional and longitudinal designs. The included studies had to evaluate frailty syndrome and cognitive impairments among participants with heart failure. As we were interested in older adults, the search was limited to individuals >65 years of age. The search was limited to primary research articles written in English published since the year 2000.

**Results:** Of the 1,245 studies retrieved by the systematic review, 8 relevant studies were enclosed for the full-text review. Our review revealed that most studies of patients with HF demonstrated evidence of an association between greater frailty and cognitive impairment. In particular, six studies reported evidence for the significant association between higher levels of frailty and cognitive impairment in patients with heart failure. The remaining two studies failed to find an association between frailty and cognitive impairment.

**Conclusions:** The development of frailty and cognitive impairment in heart failure is particularly important because this cardiovascular disease is a common cause of both morbidity and mortality in the world. The results of this review fill the existing gap in the literature related to the identification of clinical factors linked with frailty syndrome that contribute to cognitive impairment in patients with a diagnosis of heart failure. The prevalence of overlapping frailty and cognitive impairment in patients with heart failure, therefore, necessitates a routine assessment of these components in the care of patients with cardiovascular disease.

## Introduction

Heart failure (HF) is a common age-related disease that affects as many as 64.3 million people worldwide (1–3). It is the final stage of many cardiovascular diseases (CVDs), in which there is a progressive impairment of global cardiac function associated with dysfunctional hypertrophy and apoptosis of terminally differentiated cardiac myocytes (4). The number of patients living with heart failure is increasing as a result of an aging population, global population growth, and improved survival from diagnosis (5). Among older adults, both cardiovascular disease (CVD) and frailty syndrome are prevalent and often coexist (6, 7).

Frailty syndrome often coexists with HF. The prevalence of frailty in patients with HF is as high as 50% (7). Frailty increases the risk of HF and, in patients already diagnosed with HF, contributes to increased mortality, re-hospitalization, and reduced quality of life (8). Frailty often co-occurs with advanced HF, which is also accompanied by general muscle weakness and contributes to the development of cardiac cachexia at later stages of the disease.

Frailty is not consistently defined in the literature (9). Originally, frailty was synonymous with older age (9), however it was observed that a patient's response to disease, functional status, and survival are not determined solely by age. For instance, advanced HF is a clinical example of frailty independent of the patient's age (10). Further, although frailty is correlated with age, frailty does not affect only the elderly (11). Frailty also has been equated with disability and high comorbid disease burden (9). More recently, frailty has been defined as a syndrome of weakness or reserve depletion (9).

Sarcopenia is common in frailty and is associated with high disease burden, accelerated functional decline and repeated hospitalizations (12). Thus, frailty and HF, occurring together, are associated with poorer patient-reported outcomes as well as clinical outcomes (13–15). Although frailty is associated with poor outcomes, research suggests that frailty is a dynamic process and reversible. Therefore, there may be ways to prevent, modify, and control the adverse health consequences that occur through frailty. Early identification of frailty may enable the implementation of appropriate preventive health interventions that are inexpensive and easy to incorporate yet beneficial at improving clinical and patient-reported outcomes (16).

There are many tools for measuring frailty, and the number of validated tools has been increasing significantly over recent years (17). In the absence of a universal definition, measurement of frailty has been challenging. Fried et al. (9) proposed measuring frailty based on five phenotypic criteria: (1) low grip strength, (2) low energy, (3) slowness, (4) low physical activity, and/or (5) unintentional weight loss (9). According to these criteria, a person is considered frail if the patient meets at least three of the criteria listed above, and as precariously frail if one or two of the criteria are met (9). It is worth noting that Fried's criteria, while appearing to be objective and widely used by researchers and clinical practitioners around the world, refer only to physical weakness and do not include other important domains of this condition, such as cognitive weakness, psychological weakness, or social weakness (9, 18).

Although muscle strength and walking speed are impaired in frailty, cognitive function also is often affected (19). Cognitive function includes many separate domains responsible for different aspects of cognition and include memory, attention, language, psychomotor function, and executive function (20). Memory appears to be affected in frailty syndrome and is associated with increased risk of mortality (19).

Some studies have begun to include assessment of cognitive function as part of frailty diagnosis (21, 22). Remarkably, cross-sectional studies show an association between frailty and cognitive performance (9). Furthermore, longitudinal studies show an association between frailty and the onset of cognitive change, cognitive impairment, and dementia (23, 24).

Although frailty and cognitive impairment have been associated in the general population, the specific association in the HF population has not been established. People with HF are at increased risk for frailty, which can worsen symptoms, impede self-management and reduce compliance with therapy (25). Cognitive impairment is similarly prevalent in HF and leads to poorer HF outcomes (26). As both frailty and cognitive impairment are common in HF, and as both conditions are associated with poor clinical outcomes, It is important to determine if the two conditions are interrelated. If an association between frailty and cognitive impairment is identified, clinicians should be aware of the potential negative effects on clinical outcomes. Furthermore, researchers can begin to develop interventions designed to improve frailty and cognitive impairment and determine if improvement is associated with better clinical outcomes (27).

Although several studies on the association between frailty and cognitive impairment in HF have been completed, a summary of the existing evidence on the association between frailty and cognitive impairment in HF has not been completed. Therefore, the aim of this study was to complete a comprehensive review of the literature to describe the overall association between frailty and cognitive impairment in elderly patients with HF and to identify areas in need of research to better understand the association.

## Methods

This review followed the method described by Webb and Roe (28). Two teams searched four databases for studies evaluating the association between frailty and cognitive function in patients with heart failure: one team (KF, IU, ML) searched PubMed/MEDLINE and Scopus while the other (EC, TC, RS) searched Excerpta Medica database (EMBASE) and the Cumulative Index to Nursing and Allied Health Literature (CINAHL). Keywords included “heart failure,” “frail^*^ AND “cognit^*^.” The “wild card” was used to ensure that words that included the stem “frail” (frail, frailty, etc.) and “cognit” (cognitive, cognition, etc.) would be included in the search. As we were interested in older adults, the search was limited to persons >65 years of age. The search was limited to primary research articles written in English published since the year 2000. Studies evaluating the association between frailty and dementia were excluded.

The titles of the studies retrieved during the initial search were screened to determine relevance. Following initial screening, the full text of the remaining articles were read to determine if they should be included. Reviews, letters to the editor, study protocols, commentaries, clinical guidelines, and duplicates were excluded. Studies also were excluded if they did not evaluate the association between frailty and cognitive function or if they evaluated the association between frailty and cognitive function in a population other than heart failure patients. Questions about whether an article should be included were resolved by asking the advice of other members of the team. Final decisions about inclusion were determined by consensus of the team.

Once articles were identified, data were extracted and entered into a table of evidence for ease of review. Extracted data included sample size and socio-demographic characteristics, study design, measure of frailty, measure of cognitive function, and findings regarding the association between frailty and cognitive function. Questions about the information to be included in the table of evidence were resolved by discussing the matter with other members of the team and coming to consensus.

The table of evidence was reviewed to identify trends across studies. The effect of frailty on cognitive function was reviewed first. Then, trends in sample characteristics, study design, and methods of measuring frailty and cognitive function were considered. Findings and implications were discussed at weekly meetings attended by all six co-authors via Microsoft Teams. All procedures and methods performed in this review were in accordance with the ethical standards of the institutional and/or national research committee and with the 1964 Helsinki declaration and its later amendments or comparable ethical standards.

## Results

**Figure 1:**
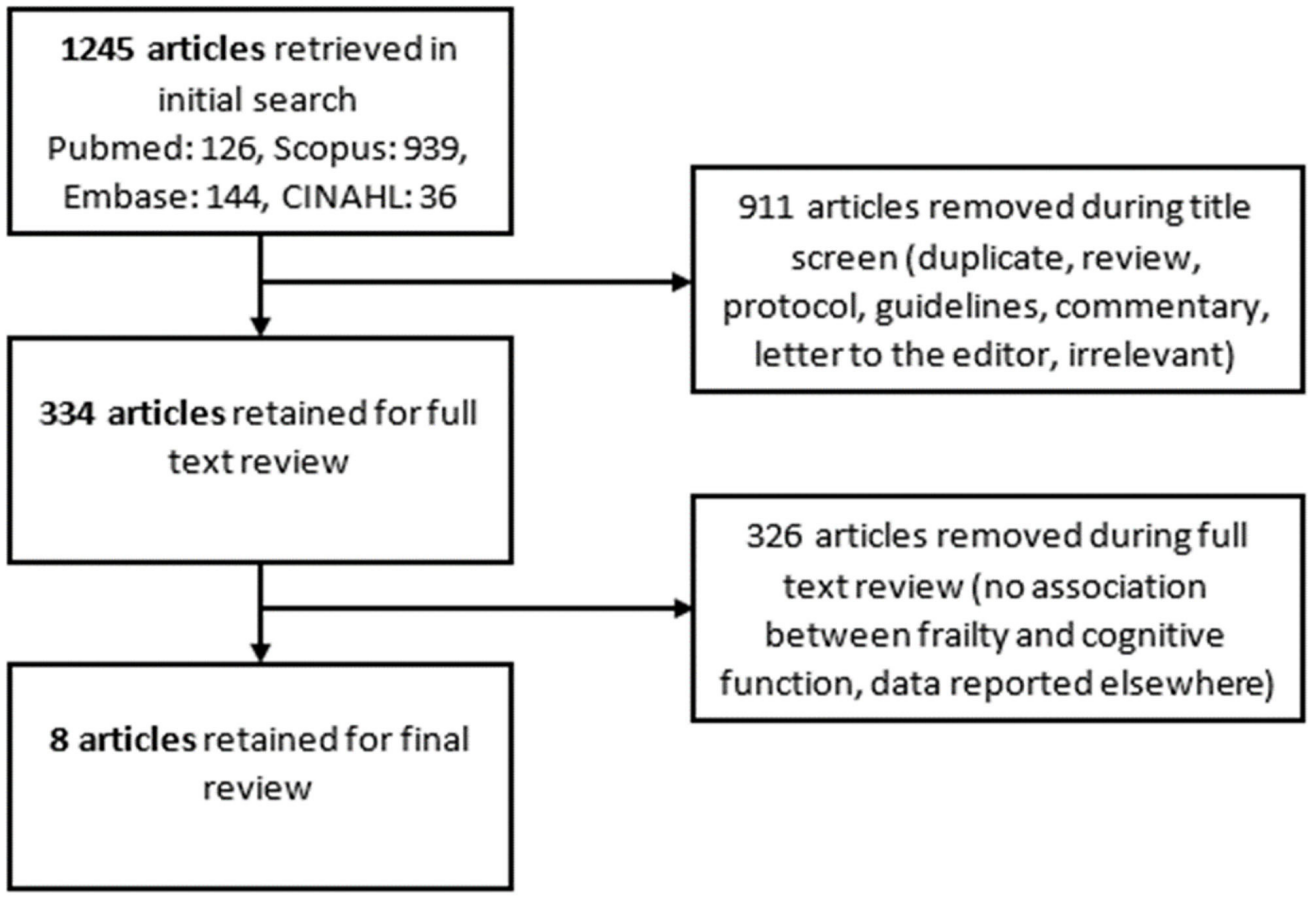
Preferred reporting items for systematic reviews and meta-analyses (PRISMA) flowchart outlining the search for relevant literature for this integrative review.

A flowchart demonstrating the literature review is presented in Figure 1 (29). After including all search terms and applying limitations, 1,245 articles were retrieved from the four databases: 126 articles from Pubmed, 939 from Scopus, 144 from Embase, and 36 from CINAHL. Following removal of 911 articles during title screening, 334 articles remained for full text review. After reading these articles, another 326 were excluded as the data had been reported in other articles or they did not report on the variables or associations of interest. Eight studies remained for inclusion in this review (30–37).

### Frailty and Cognitive Impairment

Six studies reported an association between higher levels of frailty and cognitive impairment (30–35, 37). In a small (*n* = 49) study of patients with HF, Denfeld et al. reported that cognitive impairment was more prevalent among patients with frailty (58%) than among those without frailty (8%, *p* < 0.001) (31). In a larger study (*n* = 120), Cacciatore and colleagues reported that greater frailty was associated with lower scores on the Mini Mental State Examination (MMSE) (30).

Two studies failed to find an association between frailty and cognitive impairment (33, 36). Gunton and colleagues reported no difference in cognitive function among those with high or low levels of frailty (*p* = 0.12) (33). González-Moneo et al. identified an initial association between frailty and greater odds of cognitive impairment (OR 1.58, 95% CI [1.02, 2.46], *p* = 0.04), however this association lost significance after controlling for socio-demographics and clinical measures (OR 1.58, 95% CI [0.99, 2.50], *p* = 0.05).

### Measures of Frailty

Five studies used Fried's frailty criteria as a measure of frailty (31, 34–37). Fried's frailty criteria is a well-validated indicator of physical frailty (9). Five physical criteria (shrinking, weakness, slowness, physical exhaustion, and low physical activity) are measured and then scored based on whether the measure falls above or below a given criterion. Four of the five studies using Fried's frailty criteria provided evidence of an association between frailty and cognitive impairment (31, 34, 35, 37). Joyce and colleagues reported that 15.2% of patients with frailty defined by Fried's frailty criteria also had cognitive impairment (34). Maurer et al. failed to find an association between Fried's frailty criteria and cognitive function, however this research team looked specifically at one cognitive domain rather than global cognitive function, which may explain the discrepancy (36).

Cacciatore et al. used the Frailty Staging System to rank participants based on degree of frailty (30, 38). Similar to the Fried criteria, the Frailty Staging System evaluates seven physical domains. Participants are awarded a point if the domain is intact. Higher scores indicate better function. There was evidence of an association between higher frailty stage and cognitive impairment in this study (*p* < 0.001)(30).

Gonzalez-Moneo evaluated frailty using the Barber questionnaire (32, 39). Whereas the Fried criteria and Frailty Staging System focus on physical frailty, the Barber questionnaire covers physical, psychological and social frailty (39). Although initial analysis revealed a significant association between cognitive impairment and frailty as indicated by the Barber questionnaire, the association lost significance in fully adjusted models (32). The lack of focus on the physical domain of frailty may explain the difference in the findings.

One study used a researcher-developed doorbell test as a proxy for frailty (33). Participants whose doorbell answering time fell above the median were considered more frail and those whose doorbell answering time fell below the mean were considered less frail (33). No significant difference in the proportion of people with cognitive impairment (Montreal Cognitive Impairment score <24) was observed between the two groups (*p* = 0.12), however these researchers used a researcher-developed indicator of frailty that has not been validated (33).

### Measures of Cognitive Function

Screening measures, such as the Montreal Cognitive Assessment (MoCA) and Mini Mental State Examination (MMSE) were most commonly used to measure cognitive impairment. Three studies used the MoCA to evaluate cognitive function (31, 33, 37). The MoCA is a measure that evaluates several cognitive domains and takes ~10 min to complete. Scores range from 0 to 30 with higher scores indicating better cognitive function. One point is added to the score to correct for low levels of education (12 years or fewer). Scores <26 are considered indicative of cognitive impairment (40). Two of the research teams that evaluated cognitive function using the MoCA reported an association between greater frailty and cognitive impairment (31, 37). Gunton and colleagues reported that there was no association between frailty and MoCA scores (33). Gunton did not use a validated measure of frailty, however, which may explain the lack of association.

Two studies measured cognitive function using the Mini Mental State exam (MMSE) (30, 32). The MMSE is a measure of global cognitive function (41). The MMSE takes 5–10 min to complete (41). Scores range from 0 to 30 with higher scores indicating better global cognitive functioning function (41). Scores <24 indicate global cognitive impairment (42). Both studies that used the MMSE to measure cognitive function revealed an association between frailty and cognitive impairment. Greater frailty classification was associated with lower MMSE scores (*p* < 0.001) in the study by Cacciatore and colleagues (30).

Two studies used the Mini-Cog to evaluate cognitive function (34, 35). On the Mini-Cog, participants are given three words and asked to repeat them. Then the participants are asked to draw a clock given specific guidelines and then are asked to recall the three items they were given at the beginning of the evaluation (43, 44). Scores range from 0 to 5 with scores <2 indicating cognitive impairment (43, 44). Both of the studies that evaluated cognitive function with the MiniCog provided evidence of an association between cognitive impairment and frailty, although the proportion of frail persons who had cognitive impairment was small. Of the participants with frailty in the large (*n* = 1,180) study by Matsue and colleagues, 8% also had cognitive impairment (35).

One study used the Trail-Making Test Part B to measure executive function (20). Executive function is goal-directed behavior—the ability to identify a goal and perform what is needed to achieve that goal (20, 45). On the Trail-Making Test Part B, participants are presented with a series of circles, each of which contains a letter or number. Participants are asked to connect the circles in numerical and alphabetical sequence by alternating between numbers and letters (1-A-2-B-3-C). No association between frailty and Trail-Making Test score was identified by Maurer and colleagues (*p* = 0.61) (36). It is possible that frailty is not as closely associated with executive function as with other cognitive domains, such as memory or attention, however further research is needed to evaluate the association between frailty and specific cognitive domains.

### Research Design

Seven studies used a cross-sectional research design (30–35, 37). Five of these demonstrated an association between frailty and cognitive function (30, 31, 34, 35, 37). Screening measures of global cognitive function and validated measures of frailty were used in each of these studies. The two cross-sectional studies that failed to provide evidence of an association between frailty and cognitive function included measures of frailty that have not been validated or did not focus on physical frailty specifically (32, 33).

Only one study evaluated change in frailty and cognitive function over time (36). Maurer and colleagues reported that improvements in frailty following implantation of a ventricular assist device were not associated with improvements in cognitive function (*p* = 0.61) (36). As discussed earlier, this research team focused on one, specific cognitive domain, which may have influenced the findings. Further, the sample in the final analysis of frailty and cognitive function was very small (*n* = 13). This study should be replicated on a larger sample to better understand the association between frailty and executive function following implantation of a ventricular assist device.

## Discussion

Our review revealed that most studies of patients with HF demonstrated evidence of an association between greater frailty and cognitive impairment. Recently, the aging process has received increasing attention, with a focus on interactions between cognitive impairment and frailty syndrome necessitating this review. The elderly are a heterogeneous population, which means that with age, one could expect an increasing diversity of cognitive function, physical functioning, and aspects of social involvement (46). The coexistence of frailty and cognitive impairment has important implications in the clinical evaluation of the elderly. It is worth recalling that both frailty and cognitive impairment are known predictors of negative outcomes, such as risk of hospitalization, institutionalization, falls, and mortality (47–49). As both are known to occur in patients with HF, evaluation of both should occur on a regular basis to prevent poor clinical outcomes.

Presently, the reasons behind the association between frailty and cognitive function is unknown. The International Academy on Nutrition and Aging and the International Association of Gerontology and Geriatrics has proposed a concept known as “cognitive frailty” that represents the coexistence of physical frailty and cognitive impairment, yet no mechanism behind the association was proposed (50). It is possible that one condition may contribute to the development of the other or that both conditions share a pathophysiologic mechanism, however the lack of longitudinal research in this area makes it difficult to make any conclusions about shared biological mechanisms or causative associations.

Cognitive function includes several cognitive domains, each of which has a different purpose. Memory is the system of recording, storing, and reproducing information for future use. Attention involves selecting one perceptual object, one source of stimulation, or one topic of thought from among many possible options (51, 52). Through selection, we can focus on one stimulus or source of stimulation at the expense of others. Attention applies equally to perceptual as well as to “higher” cognitive processes, such as thinking (53). Executive functions encompass a wide range of different cognitive processes, including working memory, reasoning processes, problem solving, and planning (54). Different models of executive functions are described in varying ways, but generally defined as information processing abilities that are associated with goal-directed behavior or control of complex perceptions, especially in non-routine situations (20).

Patients with HF demonstrate numerous cognitive deficits related to memory, attention, and executive functions (55–58) and working memory (59). Miller et al. (60) compared three distinct groups of HF patients: patients with global cognitive decline, patients with memory impairment, and individuals without cognitive deficits. Analysis of the measures included in the study suggested that HF patients experienced deficits in the domains of memory, attention, executive functions, and learning (60). Although it is known that HF patients demonstrate deficits in multiple cognitive domains, most studies included in this review used a screening measure to evaluate global cognitive function rather than specific cognitive domains. Therefore, to advance knowledge, it is particularly important to conduct research not only on global cognitive function but on single cognitive domains.

Each of the aforementioned cognitive processes manifests very different functional brain activity. Neuronal areas correlated with executive function include the prefrontal cortex (especially the dorsolateral prefrontal cortex, the frontal pole region, the medial and supraorbital parts of the prefrontal cortex) but also the inferior parietal, occipital, and temporal regions (61, 62). The anatomy of attention includes many areas of the brain, including the temporoparietal junction, posterior parietal, superior frontal cortex, ventral prefrontal, superior, colliculus, and pulvinar of thalamus [(63) p. 396]. For working memory, the prefrontal cortex plays a key role, but there are many alternative views regarding neural organization of working memory (64). In contrast, the medial temporal lobe areas (hippocampal region) are responsible for memory itself [(63) p. 396].

The data obtained from the studies in our review point to a possible vascular etiology of cognitive deficits among patients with heart failure but the factors that contribute to these vascular deficits are unknown. As cognitive impairment is prevalent in persons with cardiovascular disease, it is possible that the same mechanisms that causes cardiovascular disease (atherosclerosis, etc.) also cause cognitive impairment. Frailty also is highly prevalent in the cardiovascular population, so it is possible that a shared mechanism may be responsible for all conditions. However, the nature of these associations is not established.

Only one of the included studies evaluated changes in frailty and cognitive function over time and the study was limited by small sample size and confined to one cognitive domain (36). Longitudinal research is needed to begin evaluating causal relationships and to determine if one factor influences another over time. It would be interesting to evaluate how changes in frailty influence cognitive function. Similarly, it would be interesting to determine if changes in cognitive function over time influence frailty syndrome. Once associations are identified, interventions could be developed that target one phenomenon in hopes of improving the other. However, the association between the frailty and cognitive function over time first has to be established.

Most studies selected for this review used MMSE and MoCA which are screening measures of cognitive impairment. The original purpose of MMSE is assessing cognitive functioning in an elderly hospitalized population (65). The MMSE has been recommended as a dementia screen (66, 67) and MMSE cutoffs are even used for qualification for Alzheimer's Disease (AD) treatment (68). The MMSE has been used to assess dementia among various populations such as Parkinson disease (69), delirium and memory post-delirium impairments in the stroke population (70) and elderly hospital patients (71). MMSE is also employed to monitor cognitive change during treatment in patients with depression (72). However, employing MMSE in some populations (e.g., stroke, patients with diabetes, cancer, multiple sclerosis) is questionable (65).

The MMSE is intended to screen for general cognitive decline, not to assess specific cognitive domains. Indeed, the MMSE focuses primarily on orientation (10/30 points) and language (9/30 points) but has limited ability to evaluate other cognitive domains. For example, MMSE had limited ability to detect changes in attention and processing speed among patients with diabetes (73). The studies have also shown the lack of correlation of the final score with age, education, gender and ethnic differences (67, 74–76). The studies that used the MMSE to measure cognitive function revealed an association between frailty and cognitive impairment. Greater frailty classification was associated with lower MMSE scores (30) and frailty was significantly associated with higher odds of cognitive impairment (32). However, there is evidence that the MMSE is not sensitive to mild forms of cognitive impairment (40, 41, 77), therefore the prevalence of cognitive impairment in these studies may be underestimated. Thus, MMSE may be useful for assessing severity of global cognitive dysfunctions, but may be not a sensitive tool for assessing cognitive domains in patients with mild cognitive impairments and early dementia (78). Using MMSE in research on frailty does not allow researchers to make conclusions about which specific cognitive domains are associated with frailty.

The MoCA resolves some of the limitations of the MMSE but still has limitations (40). The MoCA has the ability to assess several cognitive domains: (1) short term memory; (2) Visuospatial abilities; (3) Executive functions; (4) Attention, concentration and working memory; (5) Language and (6) Orientation to time and place (40). However, the developers of the MoCA have not recommended cut-off scores that indicate impairment in each cognitive domain so it is difficult to evaluate impairment in specific cognitive domains using a screening measure. In our review we found that one of three studies that evaluated cognitive function using the MoCA reported no association between frailty and MoCA scores (33). However, as indicated earlier, Gunton did not use a validated measure of frailty which may explain the lack of association. Further, it is possible that the lack of association is due to the use of inappropriate cutoff scores to define cognitive impairment in the sample. Although Gunton et al. employed the cutoff score recommended by the creators of the measure (40), O'Driscoll and Shaikh suggest that cultural differences (diet, education, employment, activities, living arrangements, etc.] could influence scores and that cutoff scores should be individualized based on cultural background (79) Therefore, it is possible that lack of associations between frailty and cognition results from using cutoff scores that were not sensitive enough to detect cognitive impairment in the given sample.

Neurocognitive batteries are more comprehensive tests that evaluate each cognitive domain in great detail (80). Neurocognitive batteries might include separate tests that measure the domains of memory, attention, language, orientation and executive function, for example. Although they are more comprehensive and provide a good indication about dysfunction in a specific cognitive domain, neurocognitive batteries are time-consuming to complete with many of them taking an hour or more to complete (45, 80, 81). Although neurocognitive batteries are impractical in a clinical setting, they are appropriate for research studies and would provide evidence of the association between frailty and function in specific cognitive domains.

Among crucial factors for maintaining health, reducing disease complications, and improving quality of life in patients with chronic disease are behaviors relevant to self-care and adherence (82, 83). Self-care is commonly understood as a “naturalistic decision-making process in which persons engage to maintain health and manage acute and chronic illness” (83). This implies that undertaking activities directed at self-care requires patients' cognitive ability and resources.

Cognitive impairment is frequently observed in patients with HF and negatively affects cognitive function in most domains, especially memory, learning, attention, and executive function (84–90). Moreover, poor motivation related to symptoms of depression, which are common among patients with HF, can lead to poor self-care and non-compliance with healthcare guides (91). Thus, these cognitive and emotional—motivation problems of HF patients can lead to a patient's inability to engage in effective self-care behavior. Unfortunately, the healthcare system is commonly built on the assumption that patients comply with healthcare providers' recommendations (83). As cognitive impairment interferes with the ability to follow recommendations, the combination of CI and HF is associated with increased mortality, re-hospitalization [see (92)], and poor quality of life (88).

Likewise, frailty syndrome is associated with decreased ability to perform basic activities of daily living which leads to more frequent hospitalizations and death (9). Recently, frailty has also been associated with self-care behaviors in several chronic diseases (93). However, very little is known about association between frailty and self-care behaviors in HF (94). Recently, the social aspect of frailty syndrome was found to adversely affect the ability to perform self-care in Polish elderly patients with HF (25). On the other hand, hierarchical regression analysis carried out by Son et al. (93) showed that frailty is not a significant predictor of self-care when health literacy is introduced into the model.

There also is a lack of knowledge about the combined effect of frailty and cognitive impairment on self-care behaviors. Frailty is not a relevant predictor of self-care when health literacy is controlled (93). However, cognitive decline can lower health literacy which could contribute to difficulty in obtaining healthcare services (95, 96) and difficulty performing self-care (93). To date, however, it is unclear if self-care differs among frail HF patients with cognitive impairment when compared to those without cognitive impairment. It also is not clear if self-care and QOL differ among cognitively impaired HF patients depending on whether or not they have frailty. It is possible that cognitive dysfunctions moderate the relationships between frailty and self-care behaviors, but more research is needed to elucidate this.

## Limitations

The original aim of this review was to explore the relationship between cognition and frailty in the elderly with heart failure. Even though the present work has some limitations, our scrutiny has mainly achieved this purpose in our review. The main finding from this review is that the higher levels of frailty are associated with more cognitive impairments. In addition, this review highlights the presence of limitations in the previous studies exploring the association between frailty and cognition decline.

We found that majority of studies employ the frailty measures based on the Fried criteria, which addresses only physical frailty. Only one study used a more comprehensive measure of frailty which ended up with evidence for no association between frailty and cognitive decline. This suggests the large impact of conceptual issues with frailty on measures being applied to the relationship between frailty and cognitive impairments. Our recommendation is to use a consistent, validated measure of frailty in future studies exploring the relations between cognition and frailty. Also, the measure should encompass all aspects of frailty, including physical frailty, psychological frailty, and social frailty. The standard measure of frailty may be essential for comparing findings from various studies on specific domains of cognition. Further research on the relationship between cognition and frailty should also employ culturally adapted frailty measurements to the population with heart failure cut-offs point.

Like other systematic reviews, the quality of our review depends on the number and quality of the studies included. Only four databases were searched in this review, however other relevant studies may exist in other databases. There also is a risk of publication bias, in which positive results have a better chance of being published in high-impact journals (97–99). The absence of clinical studies with negative results may affect systematic reviews and distort the picture of relationships between factors being investigated (100). We also restricted our search to studies published since the year 2000, however it is possible that studies evaluating the association between frailty and cognitive function had been published earlier (98). Overall, the quality of the included studies was low, primarily because of the small sample size. Another limitation is that most of the studies were observational; hence, causation cannot be established. Lastly, all studies included in the present review investigated populations with HF patients, which also limits the generalizability of the findings of this review to other diseases.

## Implications for Research

Our work highlights several gaps in our present knowledge in terms of frailty and cognition. A major gap is that only one study has evaluated the association between frailty and a specific cognitive domain. Researchers should begin to explore the association between frailty and cognitive function more deeply and identify which cognitive domains are most affected by frailty. Once identified, clinicians will be able to address the deficits in an effort to improve self-care.

To evaluate cognitive function in specific cognitive domains, researchers will have to use specialized measures designed to evaluate each cognitive domain. Several measures exist, including the Delayed Word Recall Test to evaluate memory (101), the Digit Symbol Substitution Test to evaluate attention (102), Boston Naming Test to evaluate language (103) and Trail Making Test Part B to evaluate executive function (20, 104). Researchers interested in exploring the effect of frailty on individual cognitive domains should create a battery of valid and reliable tests that evaluate the cognitive domains without the battery being too burdensome for research participants to complete.

Another important direction is to assess causal relationships. Although there is evidence of an association between frailty and cognitive impairment, it is unclear if one disorder precedes the other or even contributes to the development of the other. Only one longitudinal studies was identified but that one study was limited by small sample size and restricted to one cognitive domain. Future studies should examine if frailty affects cognitive function or there is the opposite direction. This knowledge would help clinicians understand the relationship between the variables and anticipate changes in functional status.

There are also important questions regarding the effects of frailty and cognitive impairment on HF outcomes. For instance, researchers should compare cognitive function in HF patients with frailty and those without frailty. Relatedly, researchers also should compare the degree of frailty in HF patients who have cognitive impairment and those who do not. Finally, although both frailty and cognitive impairment are associated with poor clinical outcomes individually, it is unknown if the combined impact of frailty and cognitive impairment influences the trajectory of HF disease outcomes. We recommend further investigation on the combined impact of frailty and cognition on important outcomes such as quality of life, stress management, severity of HF's symptoms and mortality.

## Conclusions

Our review revealed an association between frailty and cognitive impairment. Although the evidence supports the association between frailty and cognitive impairment, the nature of the association has not yet been elucidated. Much more research needs to be done to determine which cognitive domains are affected by frailty and if there is a causative association between the two variables. Research also should begin to explore the combined effect of cognitive function and frailty on outcomes, such as self-care and mortality.

## Data Availability Statement

The original contributions presented in the study are included in the article/supplementary material, further inquiries can be directed to the corresponding authors.

## Ethics Statement

All procedures and methods performed in this review were in accordance with the ethical standards of the institutional and/or national research committee and with the 1964 Helsinki declaration and its later amendments or comparable ethical standards.

## Author Contributions

One team KF, IU, and ML searched PubMed/MEDLINE and Scopus while the other EC, TC, RS searched Excerpta Medica database (EMBASE) and the Cumulative Index to Nursing and Allied Health Literature (CINAHL). KF recorded data from included studies into a table of evidence for all authors to review. Each author was assigned a portion of the manuscript and wrote the draft for their assigned section. KF revised the draft for grammar and flow. All authors reviewed, approved the final version of the manuscript, and contributed to conception and design of the study.

## Conflict of Interest

The authors declare that the research was conducted in the absence of any commercial or financial relationships that could be construed as a potential conflict of interest.
